# Perioperative and long-term survival outcomes of laparoscopic versus open hepatectomy for BCLC stage A large hepatocellular carcinoma patients in difficult segments: A two-centre, propensity score matching analysis

**DOI:** 10.3389/fonc.2023.1095357

**Published:** 2023-03-10

**Authors:** Dong-yang Ding, Lei Liu, Kong-ying Lin, Xiao-jie Gan, Xing-gang Guo, Wen-bin Ding, Da-peng Sun, Wen Li, Qi-fei Tao, Fang-ming Gu, Wei-xing Guo, Yong-yi Zeng, Wei-ping Zhou, Sheng-xian Yuan

**Affiliations:** ^1^ The Third Department of Hepatic Surgery, Eastern Hepatobiliary Surgery Hospital, Naval Medical University, Shanghai, China; ^2^ Department of Hepatopancreatobiliary Surgery, Mengchao Hepatobiliary Hospital of Fujian Medical University, Fuzhou, China; ^3^ Department of Hepatopancreatobiliary Surgery, First Affiliated Hospital of Fujian Medical University, Fuzhou, China; ^4^ Department of Hepatic Surgery VI, Eastern Hepatobiliary Surgery Hospital, Naval Medical University, Shanghai, China

**Keywords:** laparoscopic liver resection, open liver resection, BCLC A, large hepatocellular carcinoma, difficult segments, propensity score matching analysis

## Abstract

**Background:**

The differences in short- and long-term outcome between laparoscopic liver resection (LLR) and open liver resection (OLR) for BCLC stage A large hepatocellular carcinoma (HCC) in difficult segments (I, IVa, VII, VIII) remain unclear. This PSM two-centre study aimed to compare perioperative and long-term survival outcomes of LLR with OLR for this HCC.

**Methods:**

HCC patients with BCLC stage A who underwent OLR or LLR in two medical centres were enrolled in the study. PSM analysis was performed to match patients between the LLR cohort and OLR cohort. Survival was analysed based on the Kaplan–Meier method. Independent risk factors were identified by Cox regression.

**Results:**

After PSM, 35 patients remained in the LLR cohort, and 84 remained in the OLR cohort. Patients in the LLR cohort had more intraoperative blood loss (p=0.036) and shorter hospital stays after surgery (p<0.001). The LLR cohort and OLR cohort had no difference in intraoperative blood transfusion, surgical margin or postoperative short-term outcomes. The OS and RFS were not significantly different between the two cohorts. The OS and RFS of these two cohorts were not different in the subgroup analysis. Surgical margin was identified as an independent risk factor for tumour recurrence.

**Conclusion:**

For BCLC stage A large HCC patients with lesions in difficult segments, LLR was feasible and had shorter hospital stay than OLR. In addition, a surgical margin ≥1 cm could significantly decrease the recurrence probability for large HCC located in different segments without compromising short-term outcomes.

## Introduction

Hepatocellular carcinoma (HCC) is one of the most common malignant diseases with insidious onset, rapid development and poor prognosis. According to cancer statistics, HCC is thought to be the seventh most common cancer and second leading cause of cancer-related death in 2020 (1). The Barcelona Clinic Liver Cancer (BCLC) staging system has been widely applied in clinical practice and provides a clinical classification of HCC (2). Incorporating the patient’s general status, tumour status and liver function status, the BCLC strategy instructs patients with prognosis prediction and treatment recommendation. The first-line treatment for HCC patients with BCLC stage A is hepatectomy, which is the most effective treatment option (3).

Traditional open liver resection (OLR) is still the gold standard for the treatment of liver cancer, which fully exposes the incision and facilitates the operation. Laparoscopic liver resection (LLR), first reported in 1991, has been widely used by hepatobiliary surgeons and identified as a safe alternative to OLR (4). In addition, meta-analyses and large propensity score-matched (PSM) studies of OLR versus LLR for HCC have strongly suggested that LLR is associated with improved perioperative outcomes, postoperative complications and hospital stays with comparable operation times, overall survival (OS) and recurrence-free survival (RFS) (5, 6). However, LLR involving difficult segments (I, IVa, VII, VIII) is still considered complex and requires advanced expertise to operate due to its limited visualization and difficulty in bleeding control (7).

With the application of advanced techniques, the complexity of LLR for HCC in difficult segments is gradually being overcome (8). Several studies have suggested that, compared with OLR, LLR is associated with fewer complications and comparable short-term outcomes and survival for HCC in difficult segments (9–11). However, these studies mainly focused on small HCC (maximum tumour size ≤5 cm), and the OLR versus LLR for large HCC (maximum tumour size ≥5 cm) in difficult segments remains unclear. This PSM two-centre study aimed to compare perioperative and long-term survival outcomes of LLR with OLR for BCLC stage A large HCC in difficult segments.

## Methods

### Patients

In this retrospective cohort study, patients who underwent LLR or OLR were selected at two tertiary hospitals, the Eastern Hepatobiliary Surgery Hospital (EHBH) and Mengchao Hepatobiliary Hospital of Fujian Medical University (MHBHFMU), from January 2014 to August 2021. A total of 3171 HCC patients were included in this cohort, including 501 laparoscopic hepatectomy and 2670 open hepatectomy. This study was conducted in accordance with the Helsinki Declaration and approved by the Clinical Research Ethics Committees of the EHBH and MHBHFMU. Patients with BCLC stage A large HCC located in difficult segments who underwent hepatectomy were enrolled in our study. The inclusion criteria in this study were as follows: 1) cases with HCC in a difficult location (segment I, IVa, VII, VIII); 2) BCLC stage A; and 3) cases with HCC ≥5 cm. The exclusion criteria were as follows: 1) patients with vascular invasion or extrahepatic metastasis; 2) insufficient data; and 3) cases lost to follow-up. Our study divided patients into a LLR cohort and an OLR cohort based on surgical approaches. The choice of surgical approach was based on full discussion between the patient and the surgeon, in accordance with the wishes of the patient. All operations were performed by experienced surgeons.

### Preoperative assessment

As described in our previous study (12), routine preoperative assessments consisted of liver dynamic computed tomography (CT), chest CT and bone scanning, as well as serological indicators related to the disease. Multidisciplinary treatment (MDT) meetings (13) were held weekly to discuss the optimal treatment options of patients. Experts from all relevant disciplines will attend, including liver surgeons, gastroenterologists, imageologists and oncologists.

### Surgical technique

Laparoscopic hepatectomy was performed as previously reported (10, 14). When the lesions were located in segments VII and VIII, patients were placed in a supine position, and the operating table was tilted 15-45° to the left during the operation. For lesions located in segment I and the superior part of segment IV, the lithotomy position was adopted in the surgical procedure. Ultrasonic shears (harmonic scalpel; Ethicon Endo-Surgery Inc., Cincinnati, OH, USA), Cavitron ultrasonic surgical aspirator (CUSA; ValleyLab Inc., Boulder, CO, USA), and LigaSure (Valley-Lab Inc.) were applied in the parenchymal transection. Large blood vessels were ligated with Hem-O-lock clips (Teleflex Medical, Research Triangle Park, NC, USA). The liver section was haemostatic with fibrin glue sealant (Greenplast, Green Cross Corp., Seoul, Korea).

The right subcostal incision extending to the midline was applied in the OLR. Parenchymal transection was conducted with an ultrasonic scalpel. If necessary, Pringle’s manoeuvre and infrahepatic vena cava clamping were used.

### Follow-up and study outcomes

In the first year after discharge, all patients were followed up every 2 months and then every 3 months until death or loss to follow-up. Routine postoperative follow-up consisted of liver dynamic CT, chest CT, and laboratory indices. HCC recurrence was identified according to CT and elevated serum AFP levels.

OS and RFS were the primary outcomes. Secondary outcomes were intraoperative outcomes (intraoperative blood loss; intraoperative blood transfusion; surgical margin) and postoperative short-term outcomes (hospital stay after surgery; 30-day mortality; postoperative complications).

### Definitions

Bile leakage was defined based on the International Study Group of Liver Surgery (15). Postoperative daily abdominal drainage of more than 10 ml/kg was defined as ascites (16). Fluid in the thoracic cavity with atelectasis requiring percutaneous drainage was defined as pleural effusion. OS was defined as the time from the date of surgery to the date of death or last recorded visit. The time from the date of surgery to the date of the first diagnosis of HCC recurrence or the last follow-up was calculated as RFS.

### Statistical analysis

In this study, continuous data with a normal distribution were compared by two-sided Student’s *t* tests, while continuous data without a normal distribution were compared using the Mann–Whitney *U* test. The chi-square test or Fisher’s exact test was adopted in the comparison of categorical data.

Propensity score matching (PSM) analysis was performed in a 7:12 matching to overcome bias in the two cohorts and balance the baseline characteristics using the nearest-neighbour matching method with no replacement. The following variables were matched: age, sex, tumour location, maximum tumour diameter, platelets, total bilirubin, alanine aminotransferase (ALT), albumin, prothrombin time, γ-glutamyl transferase, alpha fetoprotein (AFP), protein-IIinduced by vitamin K absence (PIVKA-II), hepatitis B surface antigen (HBsAg), hepatitis B virus deoxyribonucleic acid (HBV DNA), satellite nodule, tumour capsule, tumour differentiation, microvascular invasion (MVI) and liver fibrosis. We set the calliper width to 0.2 standard deviations to ensure good matching.

OS and RFS are shown by means of the Kaplan–Meier method and compared with the log rank test. Independent risk factors were identified by univariate and multivariate Cox regression. *P <*0.05 was considered statistically significant. R software (version 4.2.0, Vienna, Austria; packages: Survival and Survminer) and SPSS software (version 19.0, IBM, Armonk, New York, USA) were applied in the statistical analysis.

## Results

**Figure 1:**
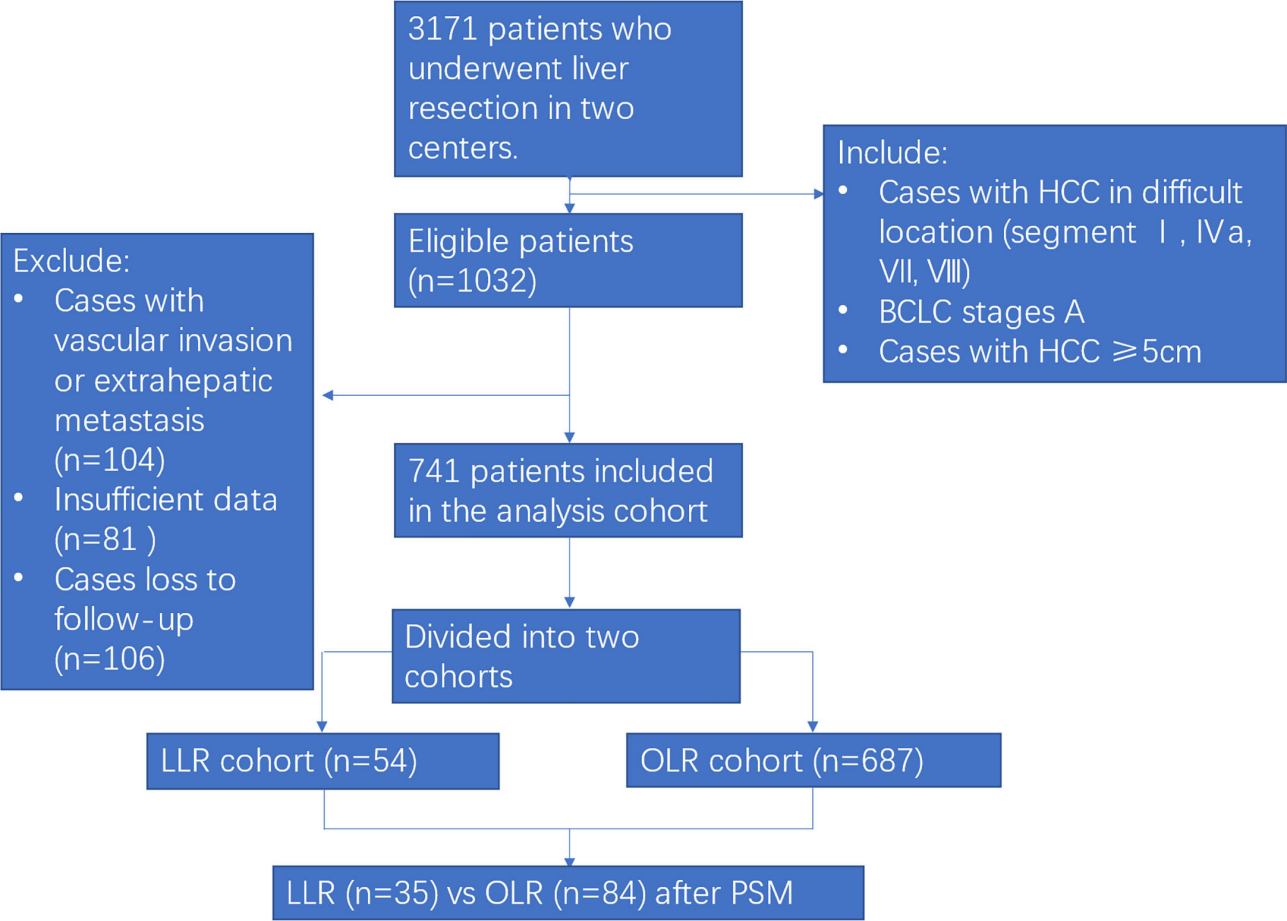
Flow chart of the study. HCC, hepatocellular carcinoma; PSM, propensity score matching; LLR, laparoscopic liver resection; OLR, open liver resection.

During the study period, 3171 patients underwent liver resection in the two centres. After excluding 104 cases with vascular invasion or extrahepatic metastasis, 81 cases with insufficient data and 106 cases lost to follow-up, 741 patients with BCLC stage A large HCC patients in difficult segments (54 patients in the LLR cohort and 687 patients in the OLR cohort) were enrolled in the analysis cohort. Finally, 119 patients (35 patients in the LLR cohort and 84 patients in the OLR cohort) were matched according to PSM analysis (**Figure 1**).

### Baseline characteristics of patients before and after PSM

**Table 1** shows the baseline characteristics of patients before and after PSM. Before PSM, compared with the OLR cohort, the patients in the LLR cohort were older (57.5 *vs*. 52, p=0.035), had a smaller tumour maximum diameter (6 *vs*. 8.1, p<0.001), lower platelet counts (164.5 *vs*. 177, p=0.039), higher levels of total bilirubin (15.8 *vs*. 12.9, p=0.014), longer prothrombin time (12.7 *vs*. 11.7, p<0.001), lower levels of PIVKA-II (1143 *vs*. 2495, p=0.03), higher percent of HBsAg positivity (88.9% *vs*. 75.1%, p=0.022), less positive Satellite nodule status (29.6% *vs*. 44.8%, p=0.036), lower degree of tumour differentiation (Grade 2 percent: 16.7% *vs*. 4.8%, p=0.005), more positive MVI status (M1 and M0: 74% *vs*. 45.4%, p<0.001) and a lower percent of liver fibrosis (40.7% *vs*. 85.2%, p<0.001). After PSM, there was no significant difference in baseline characteristics for these two cohorts, and all of the characteristics were comparable (all p>0.05) (**Table 1**).

**Table 1:** Baseline characteristics of patients before and after propensity score matching.

Variable	Before PSM			After PSM		
	LLR (n=54)	OLR (n=687)	P value*	LLR (n=35)	OLR (n=84)	P value*
Baseline characteristics						
Age (y, median (IQR))	57.5 (48,65)	52 (45,62)	**0.035**	54.3 (12.8)	55.6 (9.8)	0.558
Gender (n (%))			0.959			0.584
Female	9 (16.7)	112 (16.3)		7 (20)	13 (15.5)	
Male	45 (83.3)	575 (83.7)		28 (80)	71 (84.5)	
Tumor variables						
Tumor location (n (%))			0.806			0.735
Segment 1	0 (0)	2 (0.3)		0 (0)	1 (1.2)	
Segment 4a	7 (13)	98 (14.3)		4 (11.4)	9 (10.7)	
Segment 7	25 (46.3)	343 (49.9)		18 (51.4)	35 (41.7)	
Segment 8	22 (40.7)	244 (35.5)		13 (37.1)	39 (46.4)	
Tumor maximum diameter (cm, median (IQR))	6 (5.4,7.6)	8.1 (6.1,11.4)	**< 0.001**	6 (5.2,7.6)	6.9 (5.4,8.6)	0.108
Preoperative Blood test						
Platelets (×10^9^/L, median (IQR))	164.5 (118,194.5)	177 (139,228)	**0.039**	165 (119.5,191)	155.5 (120.8,211.8)	0.921
Total bilirubin (μmol/L, median (IQR))	15.8 (11.6,18.8)	12.9 (9.9,16.7)	**0.014**	15.5 (11.1,17.9)	12 (9.7,16.4)	0.156
ALT (IU/L, median (IQR))	38.2 (25.8,57.9)	35 (27,45)	0.296	37.7 (22.4,49.5)	35 (29.8,45)	0.9
Albumin (g/L, mean ± SD)	41.8 ± 3.4	41.3 ± 4.8	0.164	42.3 ± 5.2	41.2 ± 4	0.216
Prothrombin time (s, median (IQR))	12.7 (11.5,13.6)	11.7 (11.1,12.3)	**< 0.001**	11.9 (10.9,13.4)	11.9 (11.3,12.4)	0.755
γ-Glutamyl Transferase (IU/L, median (IQR))	78.5 (41.2,141.2)	76 (44,134)	0.709	65 (33,108.5)	76 (43,146.2)	0.212
AFP (ng/ml, median (IQR))	41.8 (6.1,1210)	155.9 (6.9,1210)	0.848	17.8 (5.4,335.5)	179.2 (14.1,1210)	0.058
PIVKA-II (mAU/mL, median (IQR))	1143 (143.2,5753.5)	2495 (1357,5701)	**0.03**	2275 (132.5,6062)	2495 (1403.2,3866.8)	0.328
HBsAg (n (%))			**0.022**			0.535
Negative	6 (11.1)	171 (24.9)		5 (14.3)	16 (19)	
Positive	48 (88.9)	516 (75.1)		30 (85.7)	68 (81)	
HBV DNA (IU/mL, median (IQR))	1235 (50,396250)	500 (50,24600)	0.326	500 (50,58150)	4780 (500,196250)	0.066
Pathology results						
Satellite nodule (n (%))			**0.036**			0.942
No	38 (70.4)	379 (55.2)		21 (60)	51 (60.7)	
Yes	16 (29.6)	308 (44.8)		14 (40)	33 (39.3)	
Tumor capsule (n (%))						0.076
None	13 (24.1)	150 (22.9)	0.29	10 (28.6)	16 (19)	
Partial	26 (48.1)	274 (39.9)		18 (51.4)	33 (39.3)	
Intact	15 (27.8)	263 (38.2)		7 (20)	35 (41.7)	
Tumor differentiation (n (%))			**0.005**			0.917
Grade 2	9 (16.7)	33 (4.8)		4 (11.4)	7 (8.3)	
Grade 3	40 (74.1)	595 (86.6)		29 (82.9)	71 (84.5)	
Grade 4	5 (9.3)	59 (8.6)		2 (5.7)	6 (7.1)	
MVI (n (%))			**< 0.001**			0.95
M0	14 (25.9)	375 (54.6)		14 (40)	31 (36.9)	
M1	18 (33.3)	245 (35.7)		12 (34.3)	30 (35.7)	
M2	22 (40.7)	67 (9.7)		9 (25.7)	23 (27.4)	
Liver fibrosis (n (%))			**< 0.001**			0.329
Yes	22 (40.7)	585 (85.2)		17 (48.6)	49 (58.3)	
No	32 (59.3)	102 (14.8)		18 (51.4)	35 (41.7)	

PSM, propensity score matching; LLR, laparoscopic liver resection; OLR, open liver resection; IQR, interquartile range; ALT, alanine aminotransferase; AFP, alpha- fetoprotein; PIVKA-II, Protein Induced by Vitamin K Absence or Antagonist-II; HBV DNA, hepatitis B virus deoxyribonucleic acid; MVI, microvascular invasion.

*P value < 0.05 is considered as statistically significant difference. The bold values denote statistical significance at P < 0.05 level.

### Long-term survival outcomes of LLR and OLR cohorts before and after PSM

**Figure 2:**
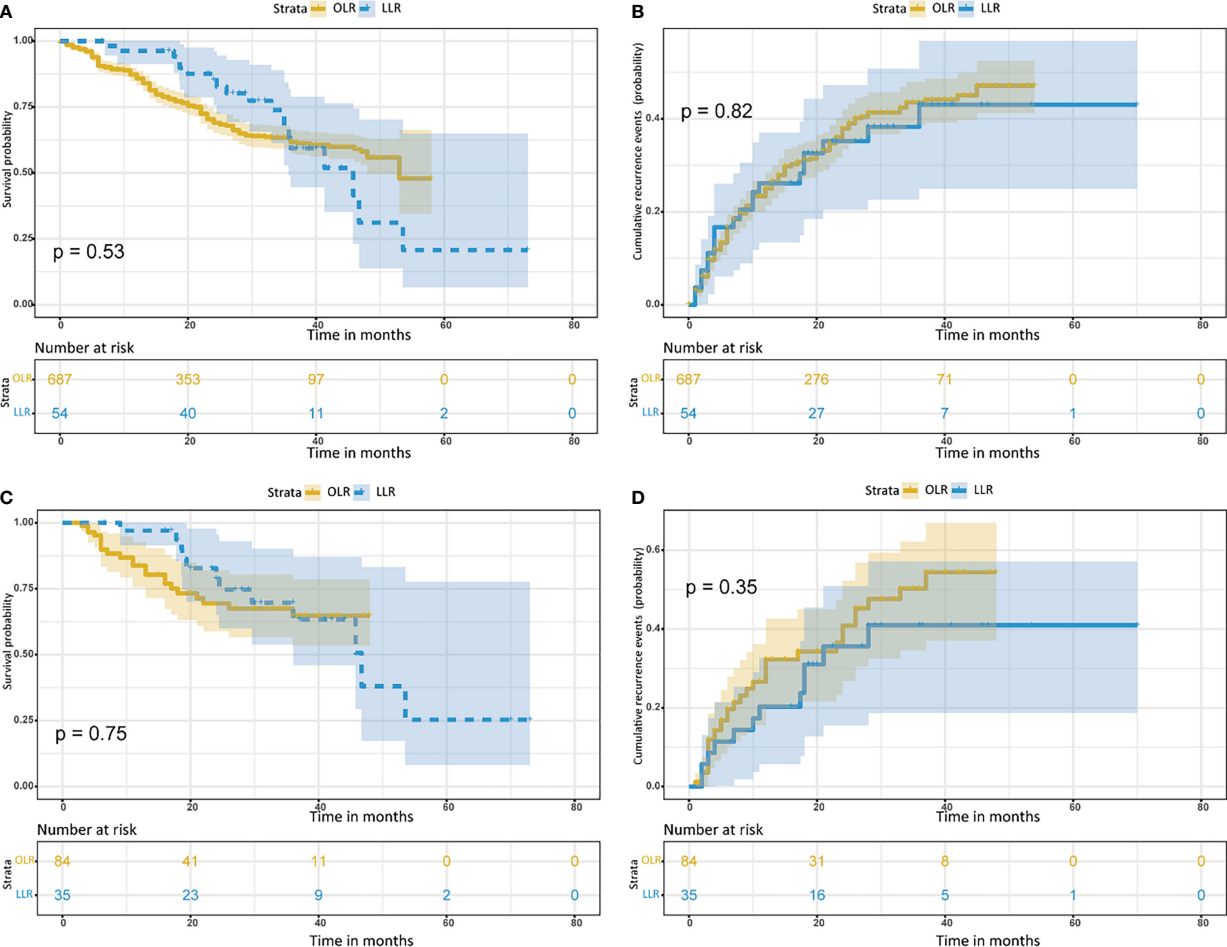
Kaplan– Meier curves for patients with hepatocellular carcinoma tumors more than 5 cm and located in the difficult segment after laparoscopic or open liver resection. **(A)** Overall survival curves and **(B)** cumulative recurrence curves before propensity score matching. **(C)** Overall survival curves and **(D)** cumulative recurrence curves after propensity score matching. LLR, laparoscopic liver resection; OLR, open liver resection.

Before PSM, the OS probabilities at 1, 2 and 3 years were 87.1%, 71.4% and 62.6%, respectively, and the cumulative recurrence probabilities at 1, 2 and 3 years were 23.4%, 36.0% and 43.5%, respectively, for the OLR cohort. In the LLR cohort, the OS probabilities at 1, 2 and 3 years were 96.2%, 87.6% and 85.2%, respectively, and the cumulative recurrence probabilities at 1, 2 and 3 years were 26.1%, 35.2% and 38.4%, respectively. These results indicated that the long-term survival outcomes between the LLR and OLR cohorts before PSM were not significantly different (both p>0.05) (**Figures 2A, B**; **Table 2**).

**Table 2:** Characteristics of intra- and postoperative outcomes and postoperative complications of patients before and after propensity score matching.

Variable	Before PSM			After PSM		
	LLR (n=54)	OLR (n=687)	P value*	LLR (n=35)	OLR (n=84)	P value*
*Intra- and postoperative outcomes:* Intraoperative blood loss (ml, median (IQR))	200 (162.5,300)	200 (100,300)	0.632	200 (200,300)	200 (100,300)	**0.036**
Intraoperative blood transfusion (n (%))			0.593			0.332
No	49 (90.7)	608 (88.5)		30 (85.7)	77 (91.7)	
Yes	5 (9.3)	79 (11.5)		5 (14.3)	7 (8.3)	
Surgical margin (n (%))						
<1cm	35 (64.8)	467 (68.0)	0.636	21 (60)	56 (66.7)	0.488
≥1cm	19 (35.2)	220 (32.0)		14 (40)	28 (33.3)	
Hospital stay after surgery (d, median (IQR))	7 (6,8)	8 (7,9)	**< 0.001**	7 (6,8)	8 (7,9)	**< 0.001**
Prognostic outcomes						
Recurrence-free (n (%))	34 (63)	458 (66.7)	0.632	23 (65.7)	52 (61.9)	0.695
Recurrence (n (%))	20 (37)	229 (33.3)		12 (34.3)	32 (38.1)	
Cumulative recurrence probability (%)			0.819			0.355
1-year	26.1	23.4		20.3	26.6	
2-year	35.2	36		35.6	36.5	
3-year	38.4	43.5		41	50.3	
Survival (n (%))	35 (64.8)	483 (70.3)	0.31	23 (65.7)	62 (73.8)	0.373
death (n (%))	19 (35.2)	204 (29.7)		12 (34.3)	22 (26.2)	
Overall survival probability (%)			0.643			0.753
1-year	96.2	87.1		97.1	83.8	
2-year	87.6	71.4		82.8	69.5	
3-year	85.2	62.6		69.7	64.8	
30-day mortality (n (%)) *Complications:* Patient number Ascites Bile leakage Operative bleeding Pleural effusion Surgical site infection Clavien–Dindo classification Grade I–II Grade III–IV	0 (0%) —— —— —— —— —— —— —— ——	10 (1.5%) —— —— —— —— —— —— —— ——	—— —— —— —— —— —— —— ——	0 (0%) 5 (14.3%) 2 (5.7%) 1 (2.9%) 1 (2.9%) 2 (5.7%) 0 (0.0%) 3 (8.6%) 2 (5.7%)	0 (0%) 18 (21.4%) 4 (4.8%) 4 (4.8%) 3 (3.6%) 4 (4.8%) 4 (4.8%) 13 (15.8%) 5 (6.0%)	0.369 0.829 0.637 0.844 0.829 0.189 0.314 0.960

PSM, propensity score matching; LLR, laparoscopic liver resection; OLR, open liver resection; IQR, interquartile range.

*P value < 0.05 is considered as statistically significant difference. The bold values denote statistical significance at P < 0.05 level.

After PSM, the OS probability at 1, 2 and 3 years was 83.8%, 69.5% and 64.8%, respectively, and the cumulative recurrence probability at 1, 2 and 3 years was 26.6%, 36.5% and 50.3%, respectively, for the OLR cohort. In the LLR cohort, the OS probabilities at 1, 2 and 3 years were 97.1%, 82.8% and 69.7%, respectively, and the cumulative recurrence probabilities at 1, 2 and 3 years were 20.3%, 35.6% and 41.0%, respectively. These results indicated that the long-term survival outcomes between the LLR and OLR cohorts after PSM were not significantly different (both p>0.05) (**Figures 2C, D**; **Table 2**).

### Subgroup survival analysis in the LLR and OLR cohorts after PSM

**Figure 3:**
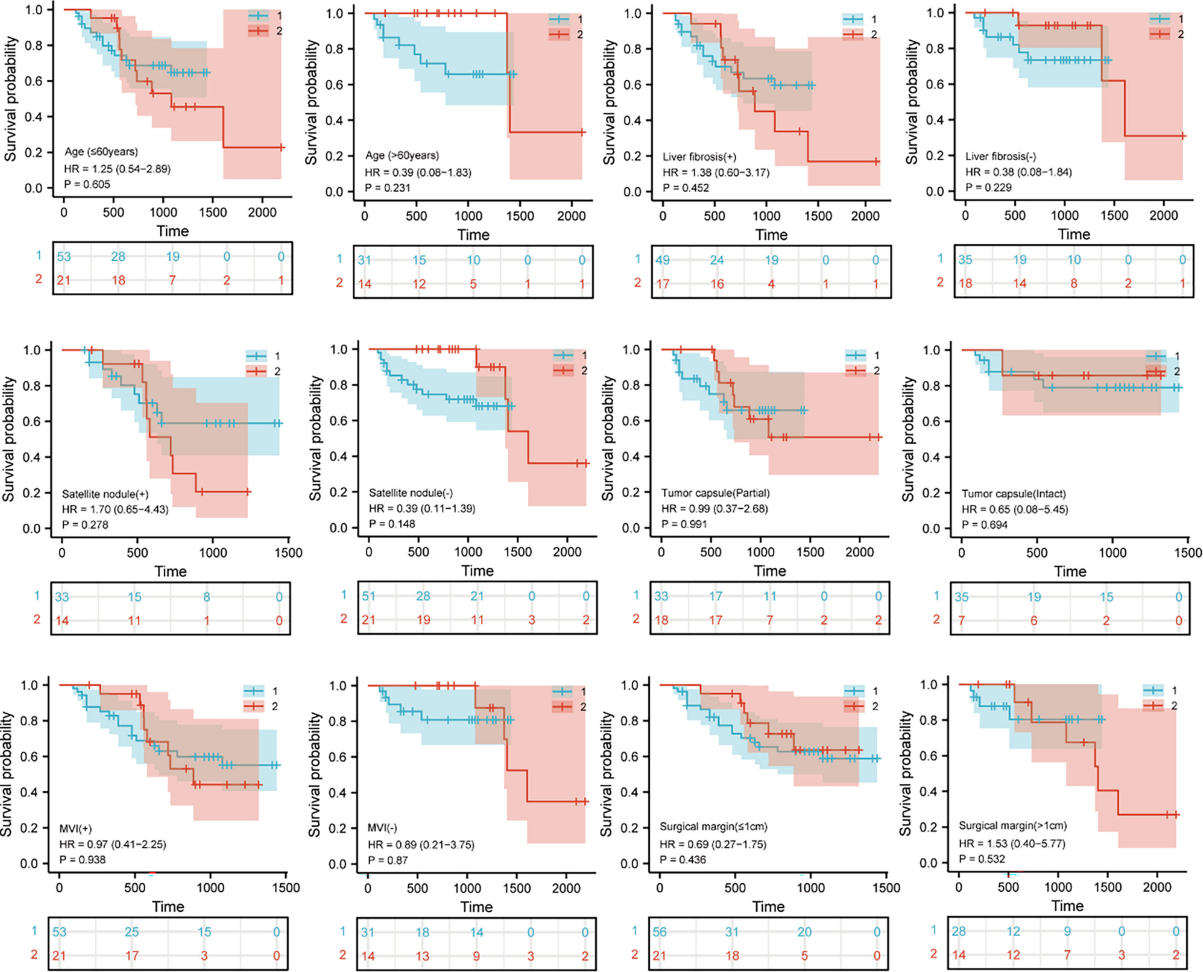
Kaplan– Meier curves for Subgroup analysis in patients with hepatocellular carcinoma tumors more than 5 cm and located in the difficult segment after laparoscopic or open liver resection. Overall survival curves after propensity score matching. “1” represents the open liver resection (OLR). “2” represents the laparoscopic liver resection (LLR).

Survival analysis was performed to explore whether there were survival differences in the LLR and OLR subgroups cohorts after PSM. According to the clinical characteristic features, we divided the patients into 6 subgroups, which included age (≤60 years or >60 years), liver fibrosis (+ or -), satellite nodule (+ or -), tumour capsule (partial or intact), MVI (+ or -), and surgical margin (≤1 cm or >1 cm). However, no significant differences were found between these two cohorts in OS probability and RFS probability based on the Kaplan−Meier method (all p>0.05) (**Figures 3**, **S1**).

### Characteristics of intra- and postoperative outcomes and postoperative complications of patients before and after PSM

**Table 2** shows the characteristics of intra- and postoperative outcomes of patients before and after PSM. Compared with the OLR cohort before PSM, the patients in the LLR cohort had shorter hospital stays after surgery (p<0.001). Other characteristics were comparable.

After PSM, compared with the OLR cohort, the patients in the LLR cohort had a higher first quartile (Q1) of intraoperative blood loss (p=0.036) and shorter hospital stay after surgery (p<0.001). However, there was no difference in intraoperative blood transfusion, surgical margin or prognostic outcomes between these two cohorts (all p>0.05).

Postoperative complications of patients were counted and analysed after PSM (**Table 2**). The patient percent of complications in the LLR and OLR cohorts was not different (14.3% *vs*. 21.4%, p=0.369). In addition, complications, including ascites (5.7% *vs*. 4.8%, p=0.829), bile leakage (2.9% *vs*. 4.8%, p=0.637), operative bleeding (2.9% *vs*. 3.6%, p=0.844), pleural effusion (5.7% *vs*. 4.8%, p=0.829), surgical site infection (0.0% *vs*. 4.8%, p=0.189) and the Clavien–Dindo classification of complications (grade I–II: 8.6% *vs*. 15.8%, p=0.314; grade III–IV: 5.7% *vs*. 6.0%, p=0.96), were not different between these two cohorts (**Table 2**). These results indicated that the postoperative complications of patients in the LLR and OLR cohorts after PSM were comparable.

### Univariate and multivariate analysis of independent risk factors before and after PSM

Before PSM, the univariate and multivariate analysis results indicated that maximum tumour diameter, partial and intact tumour capsules, and MVI:M1 were independent risk factors for survival (**Table S1**). Patient age, maximum tumour diameter, MVI:M2, γ-glutamyl transferase and surgical margin ≤1 cm were independent risk factors for tumour recurrence (**Table S2**).

After PSM, as shown in **Table 3**, maximum tumour diameter and the presence of satellite nodules were identified as independent risk factors for survival. In addition, HBV DNA level and surgical margin ≤1 cm were identified as independent risk factors for tumour recurrence.

**Table 3:** Univariate and multivariate analysis of relative risk factors for PSM cohort.

Variable	PSM cohort (n=119) for survival				PSM cohort (n=119) for tumor recurrence			
	HR(95%CI) (Univariate)	P value*	HR(95%CI) (Multivariate)	P value*	HR(95%CI) (Univariate)	P value*	HR(95%CI) (Multivariate)	P value*
*Surgery method (OLR vs. LLR)*	0.887(0.429, 1.832)	0.745	1.035(0.492,2.18)	0.928	0.730(0.375,1.421)	0.354	0.795(0.386,1.638)	0.534
Age (y)					0.972(0.946,0.999)	0.044	0.988(0.960,1.018)	0.449
Tumor maximum diameter (cm)	1.176(1.031,1.342)	0.016	**1.167(1.201,1.335)**	**0.024**				
γ-Glutamyl Transferase (IU/L)					1.002(1,1.003)	0.015	1.002(1.000,1.003)	0.096
AFP (ng/ml)					1(1,1)	0.013	1(1,1)	0.076
HBV DNA (IU/mL)					1(1,1)	0.022	**1(1,1)**	**0.029**
Satellite nodule (No *vs*. Yes)	2.223(1.11,4.452)	0.024	**2.150(1.070,4.320)**	**0.032**	2.49(1.356,4.571)	0.003	1.732(0.811,3.697)	0.156
MVI								0.95
M0 *vs*. M1					2.876(1.302,6.364)	0.009	1.593(0.641,3.961)	0.316
M0 *vs*. M2					1.442(1.896,9.428)	<0.001	1.868(0.691,5.050)	0.218
Surgical margin (<1cm *vs*. ≥1cm)					0.233(0.098,0.552)	<0.001	**0.346(0.139,0.860)**	**0.022**

PSM, propensity score matching; LLR, laparoscopic liver resection; OLR, open liver resection; AFP, alpha- fetoprotein; HBV DNA, hepatitis B virus deoxyribonucleic acid; MVI, microvascular invasion.

*P value < 0.05 is considered as statistically significant difference. The bold values denote statistical significance at P < 0.05 level.

Considering that surgical margin is an independent risk factor for tumour recurrence, which is the solely controllable variable during the operation, the long-term survival outcomes and intra- and postoperative short-term outcomes of patients with different surgical margins were compared. As shown in **Figure S2**, no significant differences were found between surgical margins ≥1 cm and <1 cm in survival probability based on the Kaplan−Meier method before or after PSM (both p>0.05). However, the RFS probability in the surgical margin ≥1 cm group before and after PSM was significantly better than that in the surgical margin <1 cm group (both p<0.001). In the comparison of intra- and postoperative short-term outcomes, the results showed that these two groups were well balanced (**Table S3**).

## Discussion

In recent decades, LLR in difficult segments has remained complex and limited due to insufficient experience, limited visualization, difficulty in bleeding control and assessment of resection margins under ultrasound. However, several retrospective studies have confirmed the safety and efficacy of LLR in these segments (9, 10). Ishizawa et al. (17) and Franken et al. (18) believe that laparoscopic hepatectomy from the I to VIII segment is safe and feasible. LLR has almost the same indications as OLR after the Second World Conference of laparoscopic hepatectomy in Morioka, Japan, in 2014 (19). Nevertheless, LLR for large HCC (maximum tumour size ≥5 cm) is still technically challenging, especially in difficult segments, and the outcomes are unclear. Although two retrospective studies have confirmed the feasibility and safety of LLR for HCC with a tumour size of 5–10 cm (20, 21), high-quality studies comparing the perioperative and oncological outcomes in BCLC stage A large HCC in difficult segments (I, IVa, VII, VIII) after LLR or OLR are still lacking. Although prospective randomized controlled trials have always been the gold standard for comparison and evaluation of therapeutic effects, it will be very difficult and sometimes even immoral to conduct such trials in clinical practice in the real world. Conversely, PSM analysis is believed to be an effective alternative to reduce selection bias and improve the level of evidence in observational comparative studies.

A previous study by V. Scuderi (10), which mainly focused on HCC ≤5 cm in posterosuperior segments (PS) of the liver, confirmed that LLR is associated with fewer complications and does not compromise survival compared with OLR in PS segments. The present study compared the perioperative and long-term survival outcomes of LLR with OLR for BCLC stage A large HCC in difficult segments. The baseline characteristics of patients in our study before PSM were different between the two cohorts, which suggested that LLR is usually performed in candidate patients. After PSM, these two cohorts were comparable.

We compared the OS probability and cumulative recurrence probability in these two cohorts before and after PSM. Patients in these two cohorts showed no significant differences in survival outcomes, and subgroup survival analysis after PSM confirmed this conclusion. In addition, we compared the characteristics of intra- and postoperative outcomes of the two cohorts before and after PSM. Although the patients in the LLR cohort had a higher first quartile (Q1) of intraoperative blood loss, the difference was not considerable. Furthermore, patients in the LLR cohort had a shorter length of hospital stay after surgery than those in the OLR cohort, and the difference was statistically significant. These results again proved that LLR for large HCC in difficult segments is feasible, safe and even more desirable.

The difficult segments are located between the liver and diaphragm, deep in the liver, which means that operation in these segments would result in right subphrenic effusion and related thoracic complications, such as pleural effusion, which prolongs the hospital stay of the patients (22, 23). In this study, postoperative complications of patients after PSM were also compared and analysed between the two cohorts. Our study results showed that the complications of the two groups were comparable, which is not consistent with previous results (9–11, 20, 21). One possible explanation for this may be that our study is the first to simultaneously incorporate the tumour maximum size ≥5 cm and tumour location in the difficult segment into the inclusion criteria. Both of these are limitations for LLR, which further increases the difficulty of surgery (24, 25). Nevertheless, LLR does not aggravate complications under the operation of our experienced surgeons compared with OLR, which confirmed the feasibility and safety of LLR for large HCC in difficult segments.

In the univariate and multivariate analyses of relative risk factors before PSM and after PSM, we found that the approaches of surgery were not independent risk factors for large HCC in difficult segments. Interestingly, surgical margin is an independent risk factor for tumour recurrence, which is the solely controllable variable during the operation depending on the surgeon. Early recurrence of HCC after hepatectomy poses a challenge to surgeons, and the effect of surgical margins is significant (26, 27). Although adequate surgical margins may be necessary to avoid early recurrence (28, 29), excessive sacrifice of the liver parenchyma to reach extensive margins results in decreased liver function and even leads to liver failure (30, 31). Consequently, surgeons should obtain surgical margins sufficient to prevent early recurrence but conservative enough to preserve the functioning liver parenchyma. In this study, we identified a surgical margin ≥1 cm as a protective factor for recurrence in large HCC located in difficult segments without compromising intra- and postoperative short-term outcomes, which is the guidance for surgeons to deal with these large malignant tumours. However, this margin is still relatively vague and needs further study for refinement.

This study has the following limitations. First, this was a retrospective study with unavoidable research bias. Although PSM analysis could minimize the difference between the LLR cohort and the OLR cohort, further randomized controlled trials should be performed to confirm the conclusions. Second, this study was conducted in China, and most patients had HBV infection (76.1%). It is not clear whether our findings can be applied to other races and aetiologies. Finally, our study was conducted in two centres. There may be some differences in patient management experience and evaluation strategies, which may have affected the reliability of the conclusions.

In conclusion, our study revealed that, for BCLC stage A large HCC patients with lesions in difficult segments, the perioperative and long-term survival outcomes of LLR were at least not inferior to those of OLR, and patients who underwent LLR had shorter recovery times than those who underwent OLR. In addition, this study defined the safe and feasible surgical margin to decrease recurrence probability for large HCC located in the different segments, which is the guidance for optimization of hepatectomy.

## Data availability statement

The raw data supporting the conclusions of this article will be made available by the authors, without undue reservation.

## Ethics statement

The studies involving human participants were reviewed and approved by Eastern Hepatobiliary Surgery Hospital (EHBH) and Mengchao Hepatobiliary Hospital of Fujian Medical University. The ethics committee waived the requirement of written informed consent for participation.

## Author contributions

D-YD, LL, K-YL and X-JG: Data curation, Writing- Original draft preparation. X-GG: Visualization, Investigation. W-BD: Software. D-PS, WL, Q-FT and FM-G: Writing- Reviewing, Editing and Revising. W-XG, Y-YZ and S-XY: Conceptualization, Methodology. W-PZ: Supervision. All authors contributed to the article and approved the submitted version.
