# Supplementary material for: Perioperative and long-term survival outcomes of laparoscopic versus open hepatectomy for BCLC stage A large hepatocellular carcinoma patients in difficult segments: A two-centre, propensity score matching analysis
Source: Front Oncol. 2023 Mar 10;13:1095357. doi: 10.3389/fonc.2023.1095357 (PMC10038276; doi:10.3389/fonc.2023.1095357)
Supplement: Supplementary file 1 [file DataSheet_1.pdf]

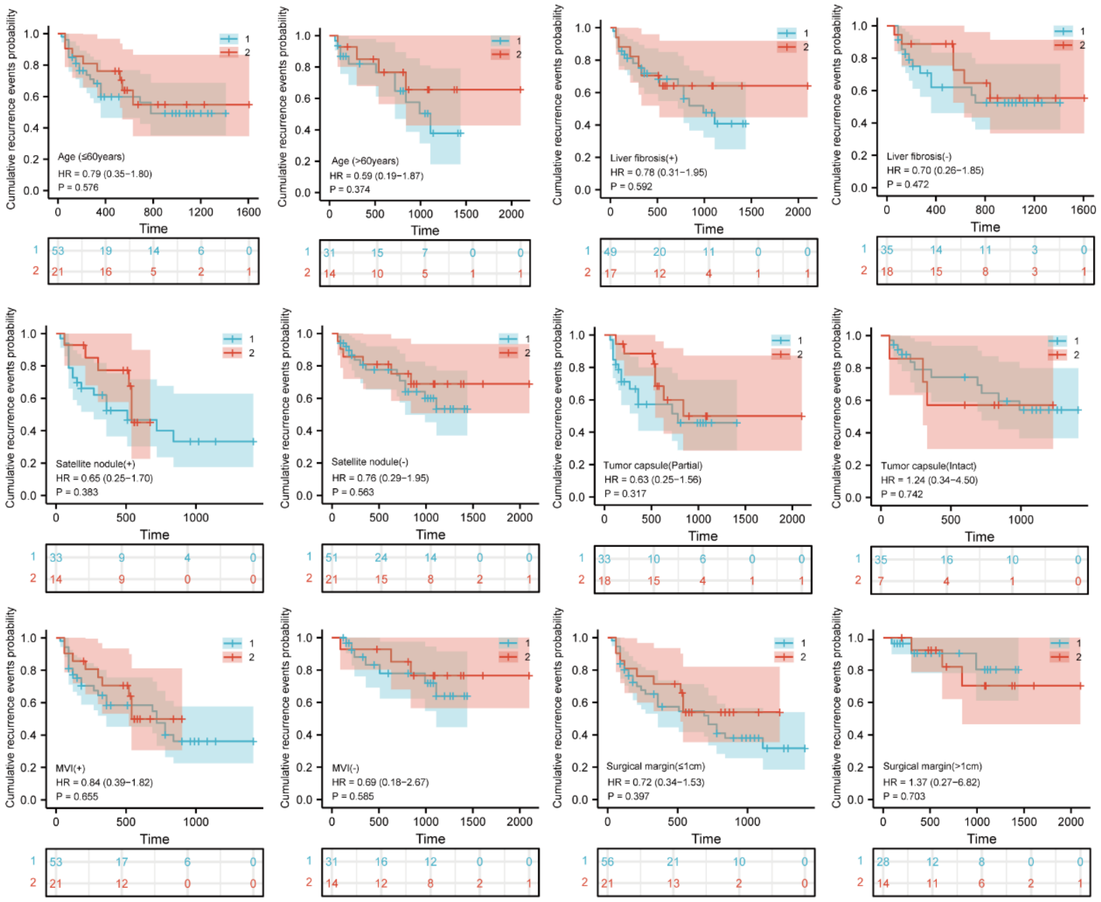

Figure S1. Kaplan– Meier curves for Subgroup analysis in patients with hepatocellular carcinoma tumors more than 5 cm and located in the difficult segment after laparoscopic or open liver resection. (A) Recurrence curves after propensity score matching. “1” represents the open liver resection (OLR). “2” represents the laparoscopic liver resection (LLR).

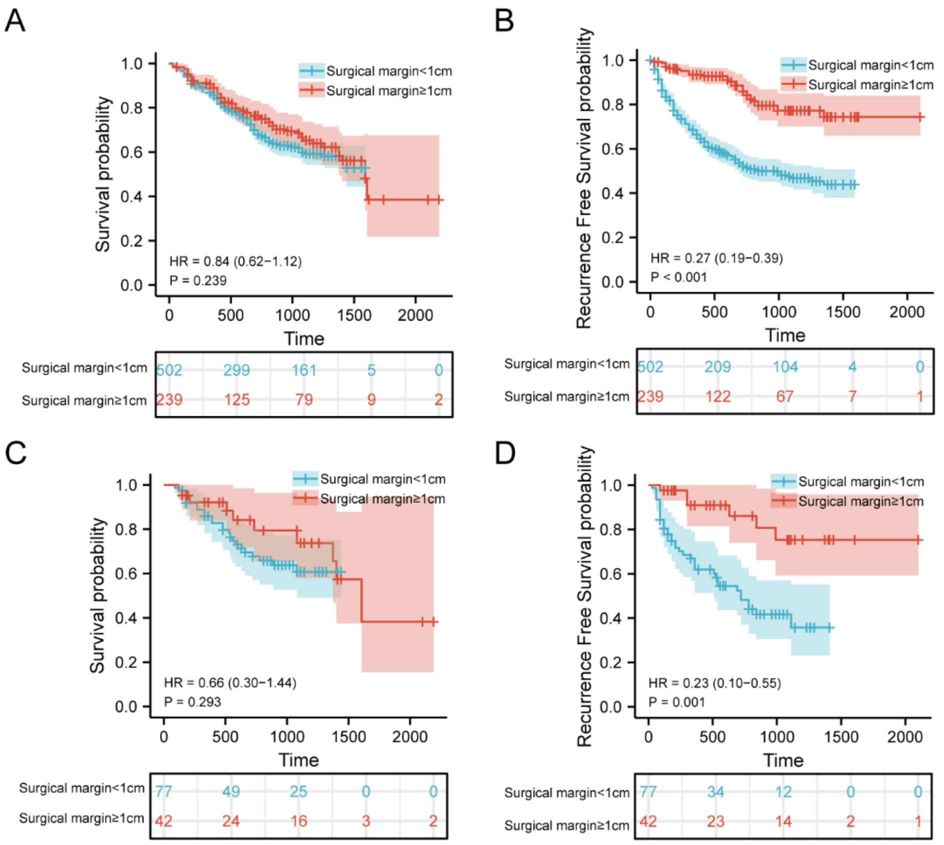

Figure S2. Kaplan– Meier curves for patients with hepatocellular carcinoma tumors more than 5 cm and located in the difficult segment in different surgical margins groups. (A) Overall survival curves and (B) Recurrence free survival curves before propensity score matching. (C) Overall survival curves and (D) Recurrence free survival curves after propensity score matching.

Table S1. Univariate and multivariate analysis of survival for patients before propensity score matching.

Characteristics	Total(N)	Univariate analysis		Multivariate analysis	
		Hazard ratio (95% CI)	P value	Hazard ratio (95% CI)	P value
Age(y)	741	0.994 (0.983-1.006)	0.313		
Gender	741				
Female	121	Reference			
Male	620	0.975 (0.682-1.393)	0.888		
Surgery method	741				
OLR	687	Reference			
LLR	54	0.855 (0.532-1.374)	0.518		
Tumor maximum diameter(cm)	741	1.106 (1.071-1.143)	**<0.001**	1.099 (1.061-1.138)	**<0.001**
Satellite nodule	741				
No	417	Reference			
Yes	324	1.375 (1.055-1.791)	**0.018**	1.120 (0.846-1.482)	0.429
Tumor capsule	741				
None	163	Reference			
Partial	300	0.688 (0.495-0.957)	**0.026**	0.636 (0.456-0.887)	**0.008**
Intact	278	0.653 (0.468-0.912)	**0.012**	0.597 (0.422-0.844)	**0.004**
Platelets (×10^9 /L)	741	1.000 (0.999-1.002)	0.643		
Total bilirubin (μmol/L)	741	1.006 (0.984-1.029)	0.583		
ALT (IU/L)	741	0.998 (0.994-1.003)	0.471		
Albumin (g/L)	741	0.981 (0.943-1.021)	0.349		
Prothrombin time (s)	737	1.060 (0.935-1.201)	0.366		

Characteristics	Total(N)	Univariate analysis		Multivariate analysis	
		Hazard ratio (95% CI)	P value	Hazard ratio (95% CI)	P value
γ-Glutamyl Transferase (IU/L)	741	1.001 (1.000-1.002)	**<0.001**	1.001 (1.000-1.001)	0.125
AFP(ng/ml)	741	1.000 (1.000-1.000)	0.242		
PIVKA-II (mAU/mL)	741	1.000 (1.000-1.000)	0.539		
HCVAb	741				
Negative	731	Reference			
Positive	10	0.276 (0.039-1.970)	0.199		
HBsAg	741				
Negative	177	Reference			
Positive	564	1.103 (0.797-1.527)	0.553		
HBV DNA(IU/mL)	740	1.000 (1.000-1.000)	0.888		
Tumor differentiation	741				
Grade 2	42	Reference			
Grade 3	635	1.592 (0.842-3.010)	0.153		
Grade 4	64	1.783 (0.852-3.732)	0.125		
MVI	741				
M0	389	Reference			
M1	263	1.651 (1.245-2.189)	**<0.001**	1.352 (1.001-1.825)	**0.049**
M2	89	1.550 (1.014-2.368)	**0.043**	1.334 (0.861-2.068)	0.197
Liver fibrosis	741				
Yes	607	Reference			
No	134	0.904 (0.635-1.286)	0.574		
Tumor location	741				

Characteristics	Total(N)	Univariate analysis		Multivariate analysis	
		Hazard ratio (95% CI)	P value	Hazard ratio (95% CI)	P value
Segment 1	2	Reference			
Segment 4a	105	0.347 (0.047-2.565)	0.300		
Segment 7	368	0.546 (0.076-3.913)	0.547		
Segment 8	266	0.429 (0.060-3.088)	0.400		
Surgical margin	741				
≤1cm	502	Reference			
>1cm	239	0.838 (0.624-1.124)	0.239		

Abbreviations: PSM, propensity score matching; LLR, laparoscopic liver resection; OLR, open liver resection; AFP, alpha- fetoprotein; HBV DNA, hepatitis B virus deoxyribonucleic acid; MVI, microvascular invasion.

*P value < 0.05 is considered as statistically significant difference.

Table S2. Univariate and multivariate analysis of recurrence for patients before and after propensity score matching.

Characteristics	Total(N)	Univariate analysis		Multivariate analysis	
		Hazard ratio (95% CI)	P value	Hazard ratio (95% CI)	P value
Age(y)	741	0.983 (0.972-0.994)	**0.002**	0.986 (0.974-0.997)	**0.015**
Gender	741				
Female	121	Reference			
Male	620	0.944 (0.677-1.316)	0.734		
Surgery method	741				
OLR	687	Reference			
LLR	54	0.948 (0.600-1.498)	0.820		
Tumor maximum diameter(cm)	741	1.067 (1.034-1.102)	**<0.001**	1.069 (1.031-1.108)	**<0.001**

Characteristics	Total(N)	Univariate analysis		Multivariate analysis	
		Hazard ratio (95% CI)	P value	Hazard ratio (95% CI)	P value
Satellite nodule	741				
No	417	Reference			
Yes	324	1.468 (1.144-1.883)	**0.003**	1.189 (0.916-1.544)	0.194
Tumor capsule	741				
None	163	Reference			
Partial	300	1.061 (0.759-1.483)	0.728		
Intact	278	1.011 (0.721-1.417)	0.950		
Platelets ($\times 10^9$ /L)	741	1.002 (1.000-1.004)	**0.019**	1.001 (0.999-1.003)	0.239
Total bilirubin (μmol/L)	741	1.012 (0.993-1.032)	0.213		
ALT (IU/L)	741	1.004 (1.001-1.006)	**0.006**	1.002 (0.999-1.005)	0.118
Albumin (g/L)	741	0.982 (0.946-1.019)	0.330		
Prothrombin time (s)	737	1.112 (0.987-1.252)	0.080		
γ-Glutamyl Transferase (IU/L)	741	1.001 (1.001-1.002)	**<0.001**	1.001 (1.000-1.001)	0.058
AFP(ng/ml)	741	1.000 (1.000-1.000)	**<0.001**	1.000 (1.000-1.000)	0.179
PIVKA-II (mAU/mL)	741	1.000 (1.000-1.000)	0.203		
HCVAb	741				
Negative	731	Reference			
Positive	10	1.033 (0.385-2.775)	0.949		
HBsAg	741				
Negative	177	Reference			
Positive	564	1.266 (0.926-1.731)	0.139		

Characteristics	Total(N)	Univariate analysis		Multivariate analysis	
		Hazard ratio (95% CI)	P value	Hazard ratio (95% CI)	P value
HBV DNA(IU/mL)	740	1.000 (1.000-1.000)	0.129		
Tumor differentiation	741				
Grade 2	42	Reference			
Grade 3	635	1.426 (0.797-2.552)	0.232		
Grade 4	64	1.702 (0.859-3.374)	0.128		
MVI	741				
M0	389	Reference			
M1	263	1.631 (1.242-2.143)	**<0.001**	1.314 (0.989-1.744)	0.059
M2	89	2.326 (1.625-3.332)	**<0.001**	1.663 (1.141-2.426)	**0.008**
Liver fibrosis	741				
Yes	607	Reference			
No	134	0.832 (0.587-1.180)	0.302		
Tumor location	741				
Segment 1	2	Reference			
Segment 4a	105	0.416 (0.057-3.029)	0.386		
Segment 7	368	0.276 (0.038-1.988)	0.201		
Segment 8	266	0.281 (0.039-2.030)	0.209		
Surgical margin	741				
≤1cm	502	Reference			
>1cm	239	0.270 (0.187-0.390)	**<0.001**	0.264 (0.182-0.384)	**<0.001**

Abbreviations: PSM, propensity score matching; LLR, laparoscopic liver resection; OLR, open liver resection; AFP, alpha- fetoprotein; HBV DNA, hepatitis B virus deoxyribonucleic acid; MVI, microvascular invasion.

*P value < 0.05 is considered as statistically significant difference.

Table S3. Intra- and postoperative short-term outcomes of patients with different surgical margins after propensity score matching

Characteristic	Surgical margin≥1cm (n=42)	Surgical margin<1cm (n=77)	P value*
Intraoperative blood transfusion (n (%))			0.536
No	39 (32.8%)	68 (57.1%)	
Yes	3 (2.5%)	9 (7.6%)	
Intraoperative blood loss (ml, median (IQR))	200 (100, 200)	200 (100, 300)	0.065
Hospital stay after surgery (d, median (IQR))	8 (7, 8)	8 (7, 9)	0.457
Complication, n (%)	11 (9.2%)	12 (10.1%)	0.247
Ascites, n (%)	3 (2.5%)	3 (2.5%)	0.664
Bile leakage, n (%)	3 (2.5%)	2 (1.7%)	0.344
Operative bleeding, n (%)	2 (1.7%)	2 (1.7%)	0.613
Pleural effusion, n (%)	3 (2.5%)	3 (2.5%)	0.664
Surgical site infection, n (%)	2 (1.7%)	2 (1.7%)	0.613

*P value < 0.05 is considered as statistically significant difference.
